# Supplementary material for: Meta-aligner: long-read alignment based on genome statistics
Source: BMC Bioinformatics. 2017 Feb 23;18:126. doi: 10.1186/s12859-017-1518-y (PMC5324271; doi:10.1186/s12859-017-1518-y)
Supplement: Additional file 1 — Supplementary file. (PDF 848 kb) [file 12859_2017_1518_MOESM1_ESM.pdf]

Supplementary Files 1
Meta-aligner

1 Analysis: Meta-aligner Alignment Stage

In this analysis, variations between target and reference genomes and also sequencing errors at individual base locations are assumed to follow i.i.d. models with rates ν and ϵ, respectively. Here, we only consider mismatch errors as the indel rate is assumed to be low enough such that the chance of observing an indel within a fragment of length ℓ_1 is sufficiently low. The probability of anchoring a given read r with i disjoint random ℓ_1-mers at the first stage of Meta-aligner algorithm is determined as follows,

$$P_a(r;i) = 1 - \left(\prod_{j=1}^{i} \left(1 - P_t(\ell_1; b_j)\right) + \sum_{k=1}^{i} P_t(\ell_1; b_k) \prod_{j=1, j\neq k}^{i} \left(1 - P_t(\ell_1; b_j)\right) \right), \tag{1}$$

where $P_t(\ell_1; b_j)$ represents the anchoring probability of an ℓ_1-mer to its true position given that the nearest repeat position is b_j bases away from the starting position of r, see Supplementary Figure 1. Note that, in Meta-aligner algorithm, since we need at least two disjoint random ℓ_1-mers for anchoring a given read, $P_a(r,0) = P_a(r,1) = 0$.

In order to determine $P_t(\ell_1; b_j)$, we let $\mathcal{A}_t(r)$ and $\mathcal{F}(r)$ denote the cases of correct and incorrect alignments, respectively. The probability of correct alignment of a fragment of length ℓ_1 with a maximum edit distance of d_{max} to its true position is,

$$\mathbb{P}\left\{\mathcal{A}_t(r)\right\} = \sum_{i \leq d_{max}} \binom{\ell_1}{i} \left(\epsilon + \nu - \frac{4\epsilon\nu}{3}\right)^i \left((1-\epsilon)(1-\nu) + \frac{\epsilon\nu}{3}\right)^{\ell_1 - i}. \tag{2}$$

Given the i.i.d. model for random intervals, such that each base is generated uniformly and independently, a fragment r of length ℓ_1 within the random interval is aligned to an incorrect position on the genome with maximum edit distance of d_{max} with the following probability (using the *union bound*),

$$\mathbb{P}\left\{\mathcal{F}(r)\right\} \leq G \times P_w(\ell_1) = G \sum_{i \leq d_{max}} \binom{\ell_1}{i} \left(\frac{3}{4}\right)^i \left(\frac{1}{4}\right)^{\ell_1 - i} = G 4^{-\ell_1} \sum_{i \leq d_{max}} \binom{\ell_1}{i} 3^i, \tag{3}$$

where $P_w(\ell_1)$ represents the probability of incorrect alignment of a fragment of length ℓ_1. Therefore, if $P_w(\ell_1)$ scales as $O\left(\frac{1}{G}\right)$, $\mathbb{P}\left\{\mathcal{F}(r)\right\}$ tends to zero and the fragment r is aligned *uniquely* to its true position with probability $\mathbb{P}\left\{\mathcal{A}_t(r)\right\}$. It can be easily verified that if $\ell_1 = 1.5 \log G$ and $d_{max} \leq 3$, $P_w(\ell_1)$ scales as $O\left(\frac{1}{G}\right)$, and therefore, all anchored reads are aligned correctly.

The next key question is what is a proper value of ℓ for human genome given its high repetitive structure. In order to answer this question, in Supplementary Figure 2, we evaluate the fraction

of random ℓ-mers for chr19 of hg19 in the case of $d = 0$, as ℓ changes from 20 to 200. The key result of this figure, is that a fragment length of $\ell = 80$ is almost sufficient to ensure that structures which appeared as repetitive for small fragment lengths, start to fully appear as random intervals. Also, as shown in Supplementary Figure 3, even for short reads of length $L = 100$, close to 98% of reads will contain random ℓ-mers if ℓ is chosen equal to 40. Since, Meta-aligner uses two ℓ-mers for anchoring, these simulations confirm that $\ell = 40$ ($\approx 1.5 \log G$ with $G = 3 \times 10^9$) is a proper choice in the case of human genome.

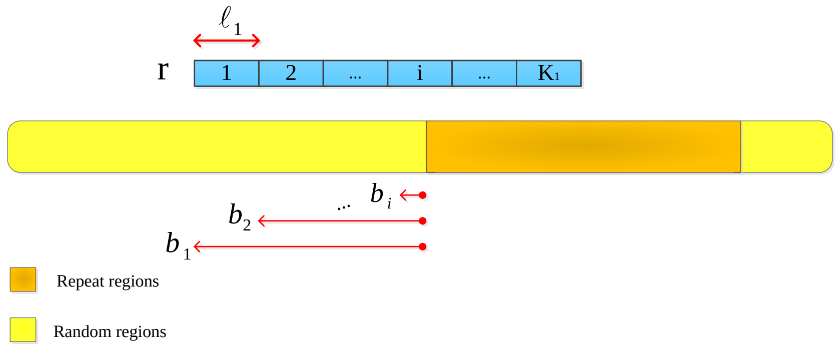

Supplementary Figure 1: **Read placement considering the genome structure**. Consider the mosaic model for a given ℓ_1 and $d = 0$, where the yellow and orange parts represent random and repeat intervals, respectively. In this figure, read r has i disjoint random ℓ_1-mers and $K_1 - i$ repeat ℓ_1-mers. The distance between j^{th} random ℓ_1-mer and the nearest repeat interval in terms of number of bases is denoted by b_j.

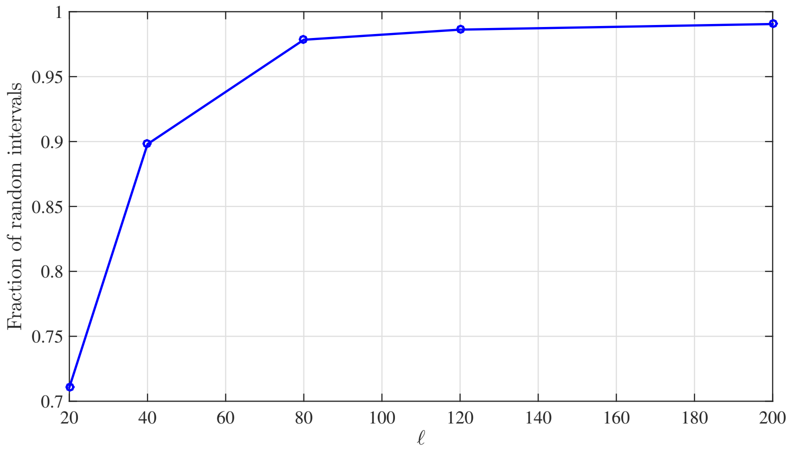

Supplementary Figure 2: **Fraction of random intervals**. Fraction of random intervals for different ℓ within chr19 of hg19 when $d = 0$.

In order to take into account the effect of repeat intervals, fragments close to these intervals (with a distance of at most ℓ_1 bases from nearest repeat interval) are multiplied by the correction term $1 - P_w(b_j)$. Thus,

$$P_t(\ell_1; b_j) \approx (1 - P_w(b_j)) \times \mathbb{P}\{\mathcal{A}_t(r)\}. \tag{4}$$

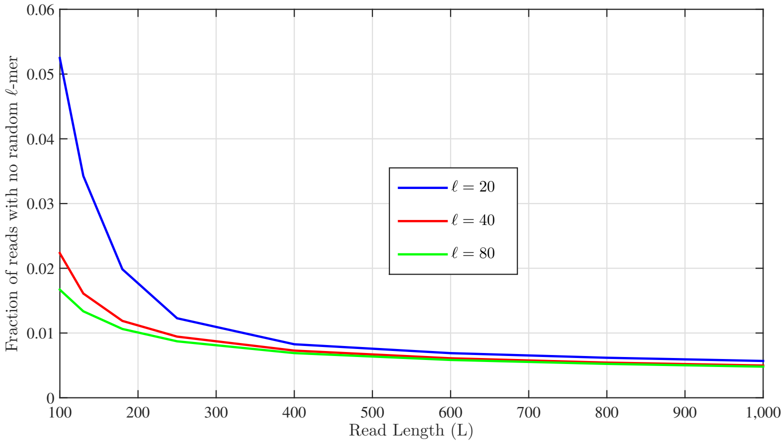

Supplementary Figure 3: **Fraction of reads with no random ℓ-mer**. This figure shows fraction of reads in chr19 of hg19 which do not have any random ℓ-mer, as a function of read length L. We consider the case of $d = 0$.

It is worth mentioning that the correction term becomes almost one for values of $b_j > 10$.

We generated synthetic reads from chr19 of hg19 with mismatch sequencing error rate of 10%. Then, using the first stage of Meta-aligner, we align each read to chr19 with $\ell_1 = 40$ and $d_{max} = 3$. Based on the proposed approach, we also determine the random and repeat intervals of chr19 in hg19 with $(\ell_1, d) = (40, 0)$. Then, we calculate (1) for this simulation. Supplementary Figure 4 shows the comparison of our analysis in (1) with simulation results. Also, in this figure results of our analysis and simulation when using sliding window with length $\mathcal{S}_1 = \ell_1/2$ are presented.

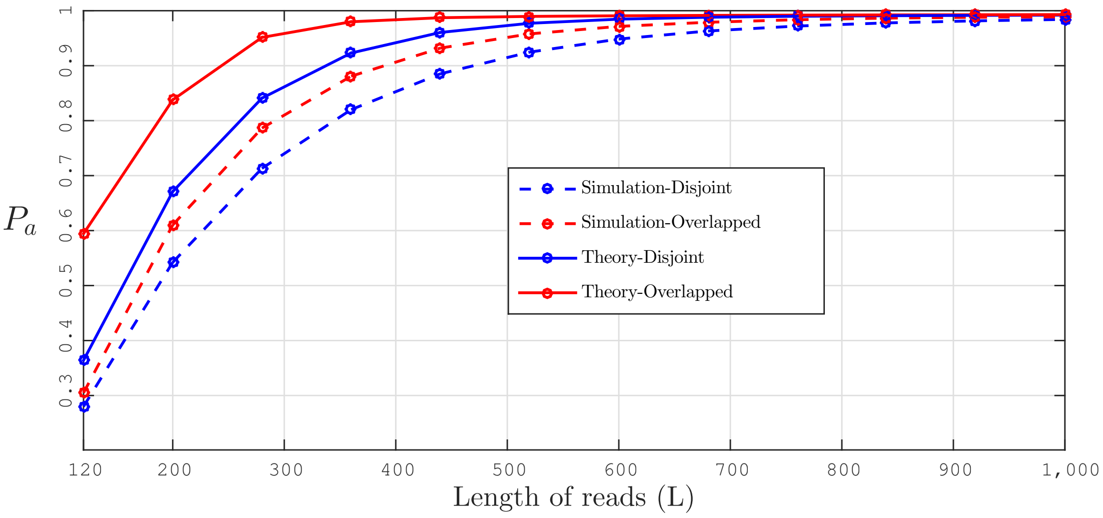

Supplementary Figure 4: **Probability of anchoring reads at the first stage of Meta-aligner**. Simulation and theoretical results are shown for chr19 of hg19 considering 10% mismatch sequencing errors.

The proposed mosaic model of the genome, for given (ℓ, d) is shown in Supplementary Figure 5. Based on this model, reads can be classified into three classes, denoted by C_1, C_2, and C_3. The classification is based on two factors: the fraction of random and repeat ℓ-mers in a read and the number of copies of an ℓ-mer. If most of ℓ-mers within a read belong to random intervals, it is classified as C_1. If most of the ℓ-mers of a read belong to repeat interval with low (resp. high) copy number then it is classified as C_2 (resp. C_3). In Supplementary Figure 6, the type of reads belonging to these classes is shown. Most of reads taken from human genome belong to C_1 and a very small fraction of reads belong to C_3. Consequently, significant improvement in speed and accuracy is achieved if an alignment algorithm aligns reads according to their classes. In fact, Meta-aligner is designed based on such concept where in its alignment stage it handles all the reads in C_1 very rapidly and accurately. Also, in the assignment stage of Meta-aligner, most of reads in C_2 are handled. For the third class, Meta-aligner applies a second round of assignment stage with Bowtie2 to handle both cases of high copy number and bad quality reads through a completely controlled level of additional complexity. In this way, Meta-aligner efficiently adapts itself to the reference genome structure and quality of reads.

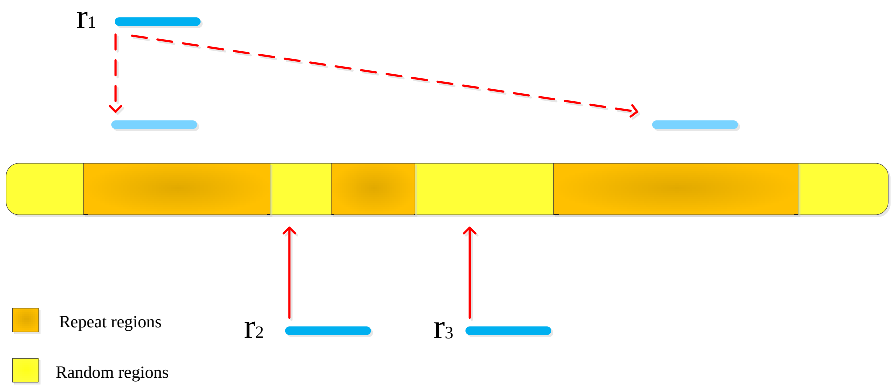

Supplementary Figure 5: **The mosaic model**. The proposed mosaic model for the genome structure, consists of two intervals, random and repeat intervals. Based on the definition of random and repeat intervals, strings that start from inside random intervals (reads r1 and r2) are uniquely mapped to the genome.

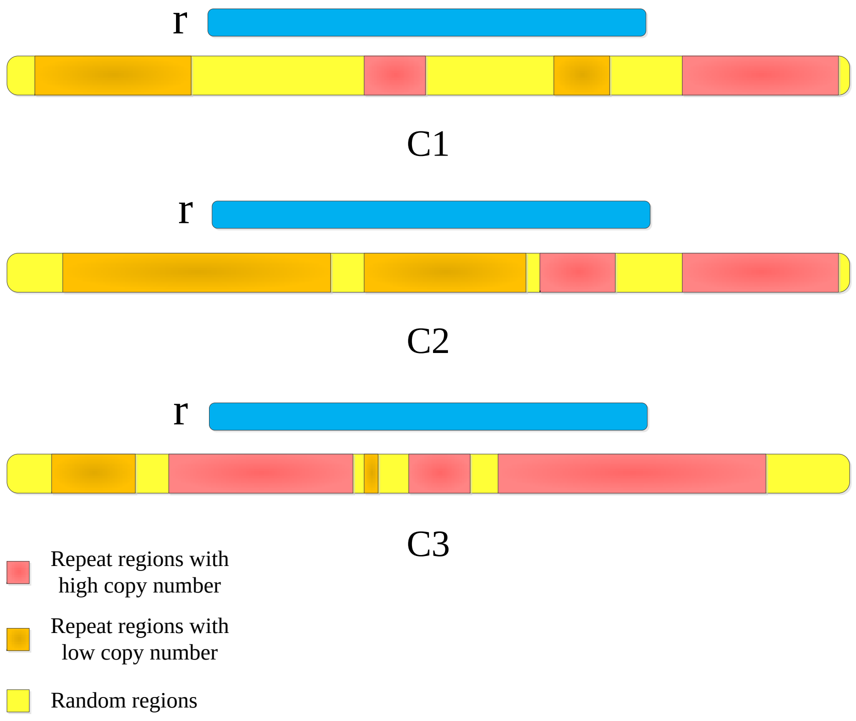

Supplementary Figure 6: **Classification of reads**. Reads are classified into three classes. Reads whose majority of ℓ-mers reside in random intervals and also the ones with a majority of ℓ-mers in low copy repeat intervals, belong to C_1 and C_2, respectively. Reads whose majority of bases are located within high copy number repeat intervals belong to C_3.

2 Details: Meta-aligner Overall Diagram and Algorithm

The details of Meta-aligner is presented through the following algorithms. In Supplementary Algorithm 1 and 2, the description of alignment and assignment procedures are presented. The path finder subroutine is presented in Supplementary Algorithm 3. The overall block diagrams of Meta-aligner and the alignment and assignment stages are shown in Supplementary Figures 7-9, respectively. The Parameter Estimation (PE) algorithm of Meta-aligner which is used for estimating ℓ, d and the slide number, is presented in the subsequent section.

Supplementary Algorithm 1 Meta-aligner: Alignment stage

Input Data: The set of N DNA reads denoted by $\mathcal{R}$. A reference genome of length G bases.
Input Aligner: Short-Read Aligner (**SRA**) $\in$ {Bowite, SOAP2, mrsFast}.
Input Parameters: ℓ_1, ℓ_2 (with $\ell_2 > \ell_1$), d_{max}, $\mathscr{G}_1$, $\mathscr{G}_2$, $\mathcal{L}_{s,1}$, $\mathcal{L}_{s,2}$, S_{th}, $\mathcal{S}_1$, $\mathcal{S}_2$ and $\#step \in \{1, 2, 3\}$.
Output: The set of aligned DNA reads denoted by $\mathcal{A}_1$.

Initiate:

for $i = 1$ to N **do**
  Set $K_i = \lfloor L_i/\ell_1 \rfloor$ for the ith read of length L_i.
  Create two arrays $[\mathscr{P}_{i,j}]_{j=1}^{K_i}$ and $[\mathscr{F}_{i,j}]_{j=1}^{K_i}$ for the ith read.
end for
Set $K_{\max} = \max_{i\in\{1,\cdots,N\}} K_i$.

---

for $h = 0$ to $\lfloor \ell_1/\mathcal{S}_1 \rfloor - 1$ **do**
  for $k = 1$ to $K_{\max}$ **do**
    Align ℓ_1 bases that started from $\{(k-1)\,\ell_1 + h\mathcal{S}_1 + 1\}$-th base of all reads in $\mathcal{R}$ with maximum distance of d_{max} using **SRA**.
    for $i = 1$ to $|\mathcal{R}|$ **do**
      if r_i Uniquely mapped **then**
        Set $\mathscr{P}_{r_i,k} = P_i$, anchored position on the genome.
        if r_i is aligned to forward strand **then**
          Set $\mathscr{F}_{r_i,k} = 1$.
        else
          Set $\mathscr{F}_{r_i,k} = 0$.
        end if
        for $j = 1$ to $k-1$ **do**
          if $|\mathscr{P}_{r_i,k} - \mathscr{P}_{r_i,j}| \le \mathscr{G}_1$ & $\mathscr{F}_{r_i,k} = \mathscr{F}_{r_i,j}$ **then**
            if $\mathscr{F}_{r_i,k} = 1$ **then**
              Add r_i with anchored position of $\mathscr{P}_{r_i,k} - (k-1)\,\ell_1 - h\mathcal{S}_1$ to $\mathcal{A}_1$.
            else
              Add r_i with anchored position of $\mathscr{P}_{r_i,k} + k\ell_1 + h\mathcal{S}_1 - L_i$ to $\mathcal{A}_1$.
            end if
            Remove r_i from $\mathcal{R}$.
          end if
        end for
      end if

end for
end for
Locally align all reads in $\mathcal{A}_1$ to their anchored positions.
Update $K_{r_i} = \lfloor \frac{L_{r_i} - h\mathcal{S}_1}{\ell_1} \rfloor$, $\forall r_i \in \mathcal{R}$.
Update $K_{\max} = \max_{r_i \in \mathcal{R}} K_{r_i}$.
end for

Supplementary Algorithm 2 Meta-aligner: Assignment stage

Input: The set of N DNA reads denoted by $\mathcal{R}$. A reference genome of length G bases.
Input Parameters: ℓ_1, ℓ_2 (with $\ell_2 > \ell_1$), d_{max}, $\mathscr{G}_1$, $\mathscr{G}_2$, $\mathcal{L}_{s,1}$, $\mathcal{L}_{s,2}$, S_{th}, $\mathcal{S}_1$, $\mathcal{S}_2$ and $\#step \in \{1, 2, 3\}$.
Output: The set of aligned DNA reads denoted by $\mathcal{A}_2$.

Initiate function:

for $i = 1$ to $|\mathcal{R}|$ **do**
Set $K_i = \lfloor (L_i - \ell_1) / \mathcal{S}_c \rfloor + 1$.
Create table $T_{i,j}(\mathcal{L}_{s,c}, \{p, s, f, ps\})$ for the ith read in $\mathcal{R}$ and $j \in \{1, \cdots, K_i\}$. $\{p, s, f, ps\}$ stands for the reported positions, scores, flags and pairs of indexes for constructing paths, respectively.
end for
Update $K_{\max} = \max_{i \in \{1, \cdots, |\mathcal{R}|\}} K_{r_i}$.
$\mathscr{A} = \emptyset$.

---

for $c = 1$ to $\#step - 1$ **do**
Run initiate function with c.
for $i = 1$ to $|\mathcal{R}|$ **do**
Add all K_i fragments of length ℓ_c for i^{th} read in $\mathcal{R}$, to $\mathscr{A}$.
end for
if c=1 **then**
Align all fragments in $\mathscr{A}$ with distance d_{max} and maximum list size of $\mathcal{L}_{s,1}$ with Bowtie.
else
Align all fragments in $\mathscr{A}$ with maximum list size of $\mathcal{L}_{s,2}$ with Bowtie2.
end if
for $i = 1$ to $|\mathcal{R}|$ **do**
for $j = 1$ to K_i **do**
Put list of alignment positions of the jth fragment in $T_{i,j}(., p)$.
Put list of alignment scores of the jth fragment in $T_{i,j}(., s)$
Put list of alignment flags of the jth fragment in $T_{i,j}(., f)$.
end for

Run *Path Finder*(r_i, $\{T_{i,1}, \cdots, T_{i,K_i}\}$; $\mathscr{L}_i$).
Find the mean score (S_m) and maximum score ($S_{\max}$) of local alignment score.
end for
for $i = 1$ to $|\mathcal{R}|$ **do**
Filter all paths in $\mathscr{L}_i$ whose local alignment score is lower than $2S_m - S_{\max}$.
if $|\mathscr{L}_i| \neq 0$ **then**
Add all paths in $\mathscr{L}_i$ to $\mathcal{A}_2$.
Remove r_i from $\mathcal{R}$.
end if
end for
Update $K_i = \lfloor (L_i - \ell_c) / \mathcal{S}_c \rfloor + 1$, for $i \in \{1, \cdots, |\mathcal{R}|\}$.
Update $K_{\max} = \max_{i \in \{1, \cdots, |\mathcal{R}|\}} K_i$.
end for

Supplementary Algorithm 3 Path Finder(r_i, $\{T_{i,1}, \cdots, T_{i,K_i}\}$; $\mathscr{L}_i$)

for $j = 1$ to $K_i - 1$ **do**
for $n = 1$ to $|T_{i,j}|$ **do**
for $m = 1$ to $|T_{i,j+1}|$ **do**
if $|T_{i,j}(n,p) + \ell_c - \mathcal{S}_c - T_{i,j+1}(m,p)| \leq \mathscr{G}_1$ & $T_{i,j}(n,f) = T_{i,j+1}(m,f)$ **then**
$T_{i,j+1}(m,s) = T_{i,j+1}(m,s) + T_{i,j}(n,s)$.
$T_{i,j+1}(m,ps) = T_{i,j}(n,ps) \cup \{(j+1, m)\}$.
else
New row for $T_{i,j+1}$, with $\{p, s, f\} = \{T_{i,j}(n,p) + \ell_c - \mathcal{S}_c, T_{i,j}(n,s), T_{i,j}(n,f), T_{i,j}(n,ps)\}$.
end if
end for
end for
end for
Set $S_{\max} = \max_n T_{i,K_i}(n,s)$.
Sort T_{i,K_i} decreasingly based on scores.
Filter all T_{i,K_i}'s with score lower than $S_{\max}/2$.
Filter all paths in the T_{i,K_i}'s with at most one index.
for $n = 1$ to $|T_{i,K_i}|$ **do**
if $|(T_{i,K_i}(n,s) - T_{i,K_i}(n+1,s))/T_{i,K_i}(n+1,s)| > S_{\text{th}}$ **then**
$N = n$.
break.
end if
end for
Locally align all substrings not aligned for any path in the T_{i,K_i} $([1:N], \{p,s,f,ps\})$.
Add local alignment score of each path as its feature in T_{i,K_i}.
Assign a list $\mathscr{L}_i = T_{i,K_i}$ $([1:N], \{p,s,f,ps\})$ to the ith read.

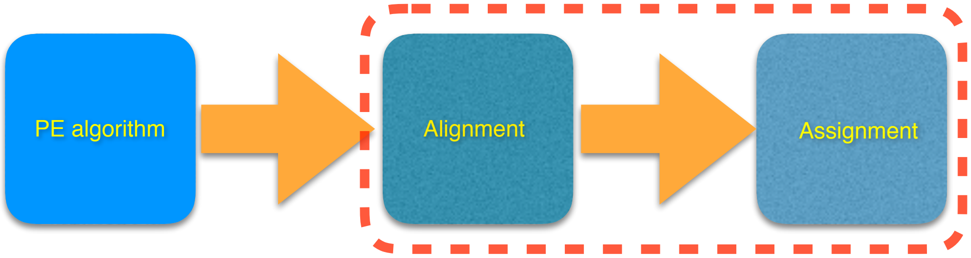

Supplementary Figure 7: **Meta-aligner overall diagram.** User can either pass input reads to the PE algorithm for estimating required parameters of Meta-aligner or adjust those parameters manually. The alignment and assignment stages can then be executed.

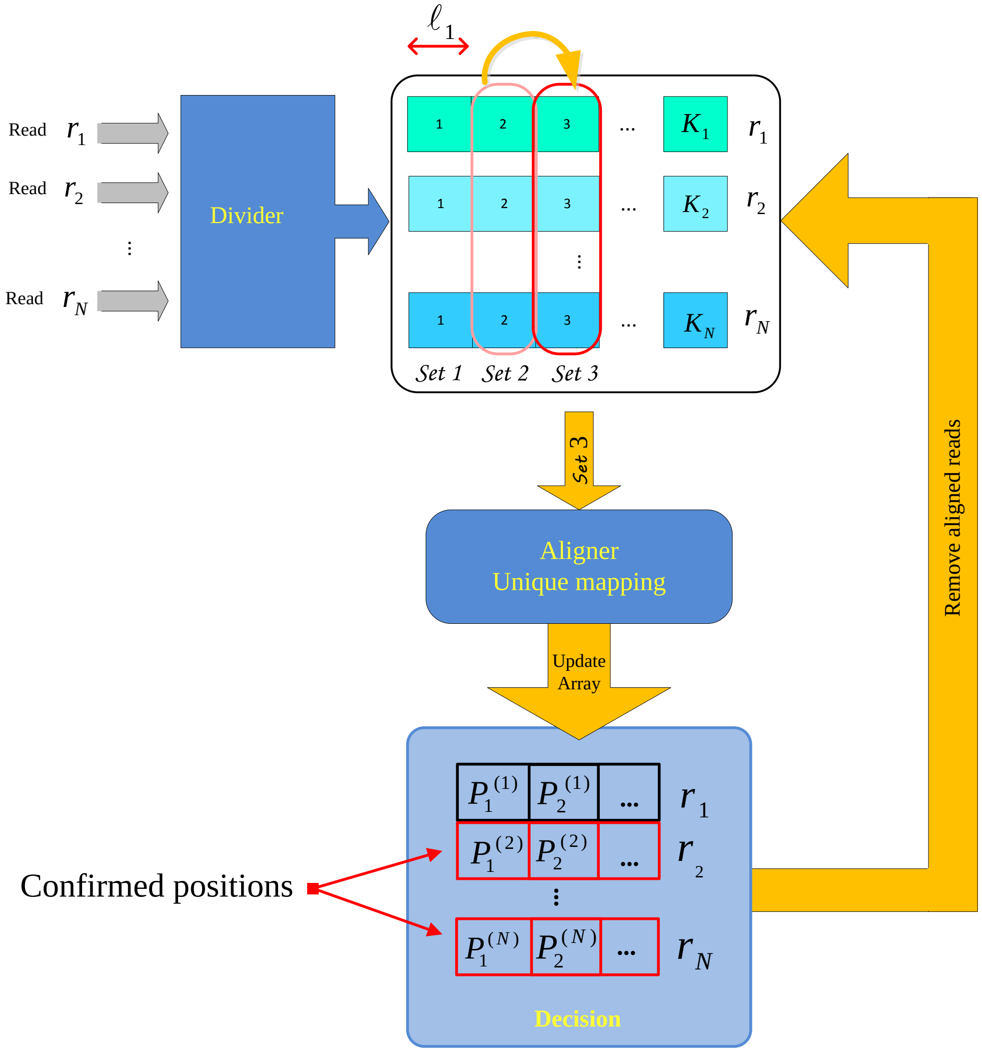

Supplementary Figure 8: **Alignment procedure.** All reads (r_i's for $i \in \{1, \cdots, N\}$) are divided into small fragments of length ℓ_1 (top block) and position of uniquely mapped fragments are stored in an array for each read (bottom block). At each stage, one set of fragments is loaded into the aligner and the position of uniquely mapped reads are stored in the array. The decision is made based on two confirming locations in the array. The read, whose location is confirmed this way, is then anchored and removed from the set of reads.

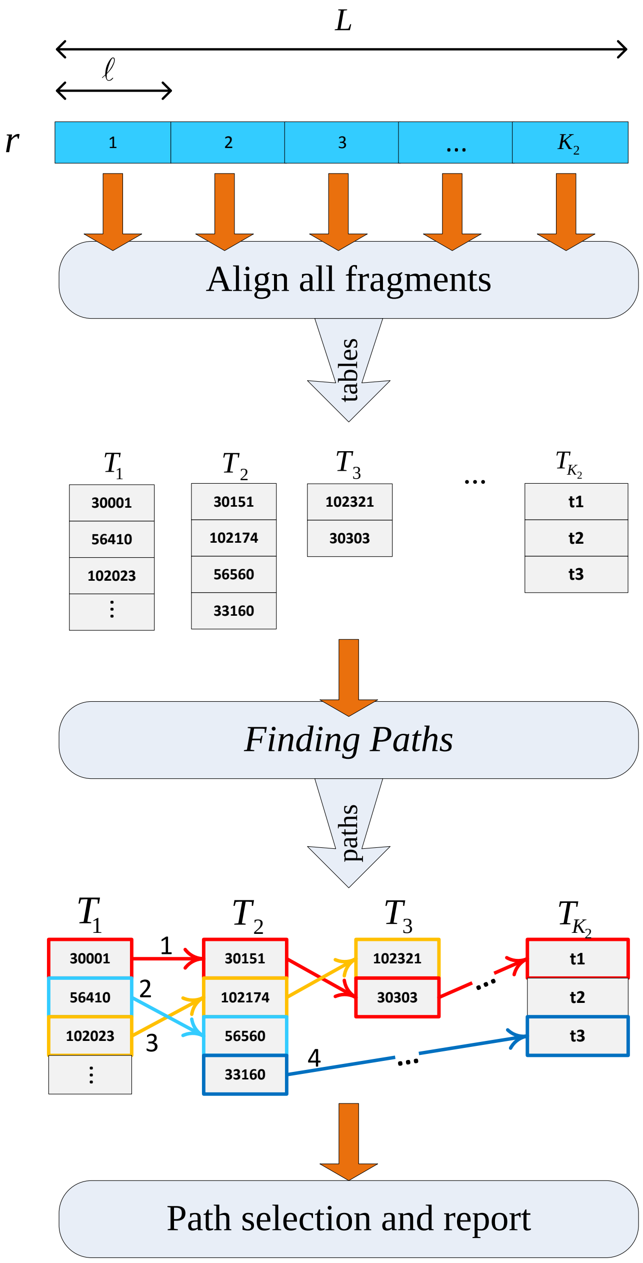

Supplementary Figure 9: **Assignment procedure.** Reads remained unaligned after the alignment procedure are divided into fragments of length ℓ (ℓ is ℓ_1 and ℓ_2 at the first and second steps of the assignment, respectively). All fragments are sent to the aligner (Bowtie or Bowtie2 depending on the step of assignment) which reports a table of positions for each fragment. All positions which confirm each other are connected and the corresponding paths are extracted. Paths may have different lengths and scores. After filtering paths, the output of the assignment stage is the list of best selected paths for a given read.

3 Description: Parameter Estimation Algorithm of Meta-aligner

Adjusting parameters of Meta-aligner, similar to what is done for other aligners, affects efficiency and quality of its performance. In order to automatically select the parameters, we propose a Parameter Estimation (PE) algorithm. This feature is of significance importance in practice, especially when no information about the input read set is available. The parameters estimated by PE algorithm are ℓ, d, slide value, and mismatch/indel sequencing error rates for local alignment adjustment. PE algorithm is presented in Supplementary Algorithm 4.

Supplementary Algorithm 4 Parameter Estimation Algorithm

Input Data: The set of N DNA reads of length $\{L_1, \cdots, L_N\}$ denoted by $\mathcal{R}$. A reference genome of length G bases.

Output: Estimated parameters: $\hat{\epsilon}_M$, $\hat{\epsilon}_G$, ℓ_1, d.

Initiate:

$\mathcal{L} = \{15, 20, 25, \ldots, 50\}$.
Set $\mathcal{T} = \emptyset$ and $L_{tot} = 0$.
while $L_{tot} < 1,000,000$ **do**
    Add the ith read (r_i) from $\mathcal{R}$ to $\mathcal{A}$.
    $L_{tot} = L_{tot} + L_i$.
end while
$R_{max} = 0$ and $T_{min} = 0$.

Run the first stage of Meta-aligner for the read set $\mathcal{A}$ with Bowtie and $\ell = 25$, $d = 2$, and without any slide.
Estimate mismatch and indel rates within reads, $\hat{\epsilon}_M$ and $\hat{\epsilon}_G$, from local alignment reports.
for $\ell \in \mathcal{L}$ **do**
    if $\lfloor (\hat{\epsilon}_M + 0.5)\,\ell \rfloor \leq 3$ **then**
        Set $d = \lfloor (\hat{\epsilon}_M + 0.5)\,\ell \rfloor$.
        Run the first stage of Meta-aligner (without local alignment) with ℓ and d.
        Save recall rate and anchoring time as $\mathcal{R}(\ell)$ and $\mathcal{T}(\ell)$.
        if $R_{max} < \mathcal{R}(\ell)$ **then**
            $R_{max} = \mathcal{R}(\ell)$ and $T_{min} = \mathcal{T}(\ell)$.
            $\ell_{op} = \ell$ and $d_{op} = d$.
        end if
    else
        remove ℓ from $\mathcal{L}$.
    end if
end for
for $\ell \in \mathcal{L}$ **do**
    if $\mathcal{R}(\ell) \geq R_{max} - 0.02$ **then**
        if $\mathcal{T}(\ell) < T_{min}$ **then**
            $\ell_{op} = \ell$ and $d_{op} = d$.
        end if
    end if
end for

---

if Average length of $\mathcal{A}$ is greater than $2,000$ **then**
    Set $s = 0$.
else if maximum recall rate is considered **then**
    if $\hat{\epsilon}_G \ell_{op} \geq 2$ **then**
        Report ℓ_{op}, $d_{op} + 1$ and slide length of 5.
        break.
    else
        Report ℓ_{op}, d_{op} and slide length of 5.
        break.
    end if
else
    Set $i = 1$, $s = 5$, $R_0 = \mathcal{R}(\ell_{op})$ and $\mathcal{T}_0 = \mathcal{T}(\ell_{op})$.
    while $\lfloor \ell_{op}/(i+1) \rfloor \geq 5$ **do**
        Run the first stage of Meta-aligner with ℓ_{op}, d_{op}, and slide value $\lfloor \ell_{op}/(i+1) \rfloor$.
        Save the recall and time as $\mathcal{R}_i$ and $\mathcal{T}_i$.
        if $(\mathcal{R}_i - \mathcal{R}_{i-1}) \leq 0.3 (\mathcal{T}_i - \mathcal{T}_{i-1})$ **then**
            Report ℓ_{op}, $d_{op} + 1$ and slide length of $s = \lfloor \ell_{op}/(i+1) \rfloor$.
            break.
        end if
        $i = i + 1$.
    end while
end if

Details of PE algorithm

We use the output of alignment stage of Meta-aligner to estimate ℓ, d, and slide value (if necessary). At the first step, we obtain an estimate of mismatch and indel rates through use of initial values of $\ell = 25$ and $d = 2$. We also select the first N_t reads of the set of input reads such that the aggregate of their lengths is greater than 1,000,000 bases. We run the first stage of the Mate-aligner for the selected reads with *Bowtie*. For the anchored reads, the local alignment reports number of mismatches and indels, denoted by n_M and n_G, respectively. Consequently, we compute, $\hat{\epsilon}_M = n_M / \sum_i L_i$ and $\hat{\epsilon}_G = n_G / \sum_i L_i$, where L_i denotes the length of the ith aligned read.

In our implementation, ℓ is selected from the set $\{15, 20, 25, 30, 35, 40, 45, 50\}$. Given $\hat{\epsilon}_M$, for any given value of ℓ, we choose $d = \lfloor (\hat{\epsilon}_M + 0.5) \ell \rfloor$ for the alignment stage. For high indel scenarios, when recall rate is significant, if $\hat{\epsilon_G} \ell \geq 2$, we change d to $d + 1$ (by considering that $d \leq 3$). The *best* pair of (ℓ, d) is selected based on its recall rate and alignment speed. Heuristically, amongst all pairs which have maximum distance of 0.02 with the best recall rate, the one whose anchoring execution time is the least is selected.

In order to estimate the slide value, we first compute the average length of the selected reads. If this average is greater than $2,000$ bps, no slide will be used. Otherwise, we run Meta-aligner with different slide numbers starting from 1 (if slide number is i, the length of slide window will be $\lfloor \ell/(i+1) \rfloor$). At the first instant that the improvement of recall rate divided by running time is less than a constant value set at $\alpha = 0.3$, we stop and report the slide value. In Supplementary Table 1, we present the results of PE algorithm for various genomes and in different scenarios. For more simulation results, see Supplementary 2.

Supplementary Table 1: The results of PE algorithm for different genomes, read lengths and sequencing error rates. Note that 90 percent of total errors (consisting of mismatch and indels) are mismatches unless specified explicitly.

Genome	L	ϵ %	$\hat{\epsilon}_M$ %	ℓ_1	d	$\mathcal{S}_1$
E. coli	300	5	4.42	35	2	-
Yeast	300	5	4.49	35	2	-
C. elegans	300	5	4.52	35	2	-
Human	300	5	4.45	30	1	-
E. coli	1,000	2	1.8	50	1	-
Yeast	1,000	2	1.8	50	1	-
C. elegans	1,000	2	1.8	50	1	-
Human	1,000	2	1.8	50	1	-
E. coli	1,000	10	8.85	40	3	-
Yeast	1,000	10	8.88	35	3	-
C. elegans	1,000	10	8.81	40	3	-
Human	1,000	10	8.85	40	3	-
E. coli	1,000	10 indel<br>10 mismatch	10.3	20	2	-
Yeast	1,000	10 indel<br>10 mismatch	10.4	20	2	-
C. elegans	1,000	10 indel<br>10 mismatch	10.39	25	3	12
Human	1,000	10 indel<br>10 mismatch	10.38	30	3	10

4 Results: Meta-aligner Incorporation of Short-Read Aligners

In the current implementation of Meta-aligner, three short-read aligners, namely Bowtie, SOAP2 and mrsFast, are used in the first stage of the algorithm. In order to compare these aligners, we generated $N = 1,000,000$ simulated reads with $\epsilon = \{2; 5; 10; 15\}$ % and different lengths $L = \{300; 1,000\}$ bps, from chr19 of hg19. Results are presented in Supplementary Table 2. For all simulations, the following parameters are reported: execution time T_1 (sec), recall rate R_1, and number of reads which are mapped incorrectly. Since the number of incorrect mappings in most cases is too low, we report the number of reads mapped incorrectly instead of precision. Once embedded in Meta-aligner, mrsFast yields the lowest number of unaligned reads except in the case of high error rate $\epsilon = 10\%$. This is due to the fact that mrsFast is initially designed to align as many as possible reads. However, mrsFast's performance is deteriorated at higher sequencing error rate scenarios, for instance, with $L = 1,000$ bps and $\epsilon = 10\%$, by using mrsFast $\approx 10,000$ errors occur, while Bowtie and Soap2 have < 100 errors. Hence, we suggest that **mrsFast** be used in cases that the the goal is achieving a **high recall rate** and the **sequencing error is low**.

SOAP2 performs almost as good as Bowtie in low sequencing error rates, with a slightly lower precision. But as the sequencing error increases, Bowtie is preferable in our algorithm's first stage and we manage to maintain a reasonable performance in terms of high recall rate, acceptable running time, and a very high precision. In addition, since Bowtie is also used in the second stage of the Meta-aligner, choosing it in the alignment stage would also be preferred if possible. Taking into account these observations, we have chosen Bowtie as the default short-read aligner for the first stage of our algorithm.

In Supplementary Table 2, the result of high indel rates is also presented. In this scenario, $L = 1,000$ bps and $\epsilon = \{10; 15\}\%$, with 10% indel rate and $\{0 \text{ or } 5\}\%$ mismatch rate. The results show very high recall and very low error in these scenarios, and both Bowtie and SOAP2 may be adopted in such setups at the first stage of Meta-aligner. As the simulation results clearly demonstrate, most of input reads can be handled at the first stage of Meta-aligner. Therefore, applications that need **only** robust and unique alignment and a high recall rate can utilize only the first stage of Meta-aligner. For high error rate scenarios, we specify the exact error rates and we use normalized cutting distance (defined as number of columns and rows that is used in the local alignment table relative to each read length) of 0.8 in the local alignment process.

Supplementary Table 2: Comparison of different short-read aligners in the first stage of Meta-aligner, for chr19 of hg19 (for read lengths of $L = \{300; 1,000\}$ bps). Note that, in the first scenario, very high error rates are presented and in the second scenario, 90 percent of total errors (that include mismatch and indel) are mismatches.

L	ϵ %	Aligner	ℓ_1	d	$\mathcal{S}_1$	R_1 (%)	Number of errors	T_1 (sec)
1,000	10 indel	Bowtie	30	2	10	88.32	90	1,897
1,000	**10 indel**	**Bowtie**	**30**	**3**	**10**	**92.36**	**253**	**2,256**
1,000	**10 indel**	**Soap2**	**30**	**3**	**10**	**95**	**107**	**1,990**
1,000	**10 indel 5 mismatch**	**Bowtie**	**30**	**3**	**5**	**91.19**	**198**	**3,196**
1,000	10 indel 5 mismatch	Bowtie	30	3	10	86.01	130	2,565
1,000	**10 indel 5 mismatch**	**Soap2**	**30**	**3**	**5**	**86.75**	**112**	**3,038**
1,000	10 indel 5 mismatch	Soap2	30	3	10	79.08	76	2,346
300	5	Bowtie	40	2	-	87.96	2	188
300	5	**Bowtie**	**30**	**2**	-	**90.32**	**4**	**201**
300	5	Bowtie	40	2	20	93.31	2	207
300	5	**Bowtie**	**30**	**2**	**15**	**93.03**	**5**	**217**
300	5	Soap2	40	2	-	93.12	9	266
300	5	**Soap2**	**30**	**2**	-	**96.42**	**27**	**273**
300	5	Soap2	40	2	20	96.54	14	287
300	5	**Soap2**	**30**	**2**	**15**	**97.56**	**37**	**284**
300	5	mrsFast	40	8	-	95.55	8915	228
300	5	**mrsFast**	**30**	**6**	-	**95.4**	**12523**	**239**
300	5	mrsFast	40	8	20	98.46	10172	258
300	5	**mrsFast**	**30**	**6**	**15**	**97.75**	**13543**	**304**
1,000	10	Bowtie	40	3	-	97.78	**19**	1,209
1,000	**10**	**Bowtie**	**30**	**3**	-	**98.37**	**54**	**1,212**
1,000	5	Bowtie	40	2	-	**99**	**3**	1,060
1,000	5	Bowtie	30	2	-	**98.89**	**12**	1,081
1,000	2	Bowtie	40	1	-	**99.27**	**0**	1,030
1,000	10	Soap2	40	3	-	93.31	31	1252
1,000	**10**	**Soap2**	**30**	**3**	-	**98.9**	**140**	**1,232**
1,000	5	Soap2	40	2	-	**99.39**	28	1,138
1,000	5	Soap2	30	2	-	**99.43**	71	1,152
1,000	2	Soap2	40	1	-	**99.52**	**8**	1,106
1,000	10	mrsFast	40	25	-	95.12	8697	1,240
1,000	10	mrsFast	30	30	-	95.98	11967	1,306
1,000	5	mrsFast	40	10	-	**100**	11070	1,126
1,000	5	mrsFast	30	15	-	**100**	14925	1,168
1,000	2	mrsFast	40	5	-	**100**	11095	1,111
1,000	2	mrsFast	30	3	-	**100**	14783	1,140

5 Results: Meta-aligner Simulation Results on the hg19

In this part, we present Meta-aligner simulation results for the human genome (hg19) using Bowtie at the first stage of the algorithm. Similar to the previous section, $N = 1,000,000$ simulated reads are generated with $\epsilon = \{2; 5; 10; 15; 20\}$ % and different lengths $L = \{300; 500; 1,000\}$ bps, from the reference genome. If not specified explicitly, the sequencing errors are 90% mismatches and 10% indels. In Supplementary Tables 4-5, the following parameters are reported for only the first stage of Meta-aligner: execution time T_1 (sec), recall rate R_1, and number of reads which are mapped incorrectly. For high error rate scenarios, we specify the exact error rates and we use normalized cutting distance of 0.8 in the local alignment. Results of the overall algorithm of the Meta-aligner (results of the first and the second steps) is presented in Supplementary Tables 3 and 6, and the following parameters are reported: time of the second step (T_2) and overall time (T_{tot}), recall rate of the second step (R_2) and overall recall rate (R_{tot}), and precision of the second step (P_2) and overall precision (P_{tot}). The simulation results reveal that the first stage of the algorithm can very rapidly and with very high precision handle most of the reads, especially for longer reads (e.g. $L \geq 1,000$ bps) even at high error rates. In applications where repeat intervals are not the main concern, by running only the first stage of the algorithm an acceptable recall rate with very high precision is achievable.

In the assignment stage when Bowtie with list size of 10 is used, many reads within the repeat intervals with low copy number are handled very fast. For this step, we consider the same parameters with the alignment stage (except for slide number whose default value is 1, i.e. length of sliding window is $\lfloor \ell_1/2 \rfloor$). Results show that these two steps complement each other. However, if one needs more recall rate (and execution time is not the highest priority) Meta-aligner has the option for running the third step, i.e. the second step of the assignment stage, with Bowtie2 using default sub-fragment and list size of 150 and 40, respectively. As mentioned earlier, this step is specifically designed for handling long repeat intervals with high copy number and very bad quality reads. Note that for high indel rates, we set normalized cutting distance of the local alignment table at 5× *indel rate*.

Supplementary Table 3: The overall Meta-aligner results for hg19 in the case of very high error rates ($\epsilon = \{15; 20\}$ %) and read lengths of $L = \{4,000; 8,000\}$ bps).

L	ϵ %	$(\ell_1, d, \mathcal{S}_1)$	R_2 (%)	P_2 (%)	T_2 (sec)	R_{tot} (%)	P_{tot} (%)	T_{tot} (sec)
4,000	**15 indel**	$(25, 2, -)$	81.44	90.41	**401**	**98.72**	**99.41**	**4,899**
4,000	**15 indel**	$(25, 2, 12)$	75.28	87	**298**	**98.78**	**99.41**	5,103
4,000	**15 indel**<br>**5 mismatch**	$(25, 2, -)$	83.97	94.41	**675**	**98.13**	**99.41**	5,435
4,000	**15 indel**<br>**5 mismatch**	$(25, 2, 12)$	73.42	90.16	**378**	**98.23**	**99.43**	5,692
8,000	**20 indel**	$(25, 2, -)$	88.46	92.49	**576**	**99.26**	**99.53**	13,283
8,000	**20 indel**	$(25, 2, 12)$	83.43	89.57	**357**	**99.34**	**99.58**	13,668
8,000	**15 indel**<br>**5 mismatch**	$(25, 2, -)$	86.88	89.83	**348**	**99.48**	**99.59**	**12,887**
8,000	**15 indel**<br>**5 mismatch**	$(25, 2, 12)$	83.82	87.67	**270**	**99.52**	**99.61**	13,170

Supplementary Table 4: The results of the first stage of Meta-aligner for hg19 at different error rates ($\epsilon = \{2; 5; 10\}$ %) and read lengths ($L = \{300; 500; 1,000\}$ bps). Note that 90 percent of total errors (that include mismatch and indel) are mismatches unless specified explicitly.

L	ϵ %	Aligner	ℓ_1	d	$\mathcal{S}_1$	R_1 (%)	Number of errorss	T_1 (sec)
1,000	10 indel	Bowtie	30	3	10	88.87	115	4,410
1,000	10 indel	Bowtie	30	2	10	87	55	2,366
**1,000**	**15 indel**	**Bowtie**	**25**	**2**	**5**	**81.76**	**108**	**5,383**
1,000	10 indel<br>5 mismatch	Bowtie	30	3	5	88.08	68	7,075
**1,000**	**10 indel**<br>**5 mismatch**	**Bowtie**	**30**	**3**	**10**	**84.23**	**30**	**5,305**
**1,000**	**10**	**Bowtie**	**40**	**3**	-	**94.1**	**19**	**1,342**
1,000	10	Bowtie	30	3	-	94.25	52	1,638
1,000	5	Bowtie	40	3	-	95.3	8	1,181
**1,000**	**5**	**Bowtie**	**40**	**2**	-	**95.88**	**12**	**1,078**
1,000	5	Bowtie	30	2	-	95.32	12	1,133
1,000	2	Bowtie	40	2	-	95.71	0	1,050
**1,000**	**2**	**Bowtie**	**40**	**1**	-	**96.74**	**1**	**1,029**
1,000	2	Bowtie	30	1	-	96.24	0	1,057
500	5	Bowtie	40	3	-	92.56	2	412
**500**	**5**	**Bowtie**	**40**	**2**	-	**93.03**	**1**	**323**
500	5	Bowtie	30	2	-	92.43	4	349
500	5	Bowtie	40	3	-	93.78	3	452
**500**	**5**	**Bowtie**	**40**	**2**	**20**	**94.44**	**1**	**347**
500	5	Bowtie	30	2	15	93.7	7	383
500	5	Bowtie	30	2	10	94.17	7	413
300	5	Bowtie	40	3	-	86.66	1	299
300	5	Bowtie	40	2	-	85.79	0	218
**300**	**5**	**Bowtie**	**30**	**2**	-	**87.42**	**2**	**243**
300	5	Bowtie	40	3	20	89.38	2	345
300	5	Bowtie	40	2	20	89.79	1	249
300	5	Bowtie	30	2	15	89.24	3	271
**300**	**5**	**Bowtie**	**30**	**2**	**10**	**90.05**	**5**	**296**

Supplementary Table 5: The first stage of the Meta-aligner results for hg19 in the case of very long reads and high error rates.

L	ϵ %	Aligner	ℓ_1	d	$\mathcal{S}_1$	R_1 (%)	Number of errors	T_1 (sec)
4,000	15 indel	Bowtie	25	2	-	93.12	70	4,498
**4,000**	**15 indel**	**Bowtie**	**25**	**2**	**12**	**95.07**	**107**	**4,805**
4,000	15 indel	Bowtie	25	2	5	96.16	143	5,426
4,000	15 indel<br>5 mismatch	Bowtie	25	2	-	88.31	30	4,760
**4,000**	**15 indel<br>5 mismatch**	**Bowtie**	**25**	**2**	**12**	**93.34**	**51**	**5,314**
4,000	15 indel<br>5 mismatch	Bowtie	25	2	5	95.34	98	6,119
8,000	20 indel	Bowtie	25	2	-	93.56	30	12,707
**8,000**	**20 indel**	**Bowtie**	**25**	**2**	**12**	**96**	**39**	**13,311**
8,000	20 indel	Bowtie	25	2	5	97.06	67	14,199
8,000	15 indel<br>5 mismatch	Bowtie	25	2	-	96	25	12,539
**8,000**	**15 indel<br>5 mismatch**	**Bowtie**	**25**	**2**	**12**	**97.01**	**40**	**12,900**
8,000	15 indel<br>5 mismatch	Bowtie	25	2	5	97.66	85	13,577

Supplementary Table 6: The overall Meta-aligner results for hg19 at different error rates ($\epsilon = \{2; 5; 10\}$ %) and read lengths ($L = \{300; 500; 1,000\}$ bps). The case of error rate of $\epsilon = 15\%$ for $L = 1,000$ bps is also shown separately. Note that 90 percent of errors are mismatches unless specified explicitly.

L	ϵ %	$(\ell_1, d, \mathcal{S}_1)$	R_2 (%)	P_2 (%)	T_2 (sec)	R_{tot} (%)	P_{tot} (%)	T_{tot} (sec)
300	5	$(40, 2, -)$	76.55	97.96	**123**	96.67	**99.76**	**341**
300	5	$(40, 2, 20)$	68.01	96.86	**92**	96.73	**99.76**	**341**
500	5	$(40, 2, -)$	78.68	96.49	**108**	**98.51**	**99.79**	**431**
500	5	$(40, 2, 20)$	73.66	95.42	**84**	**98.54**	**99.79**	**431**
1,000	2	$(40, 1, -)$	73.95	92.87	**95**	**99.15**	**99.80**	**1,124**
1,000	2	$(40, 2, -)$	56.31	90.23	125	98.13	**99.73**	**1,175**
1,000	5	$(40, 2, -)$	84.52	95.85	**132**	**99.36**	**99.84**	**1,210**
1,000	10	$(30, 3, -)$	77.75	93.69	493	**98.72**	**99.68**	2,133
1,000	10	$(40, 3, -)$	80.70	95.41	**274**	**98.86**	**99.76**	**1,616**
1,000	**10 indel**	$(30, 3, 10)$	67.06	80.61	**786**	**96.33**	**98.10**	5,196
1,000	**10 indel**	$(30, 2, 10)$	68.55	84.87	**681**	**95.91**	**98.27**	**3,047**
1,000	**15 indel**	$(25, 2, 5)$	72.72	76.88	2464	**95.02**	95.66	7,847
1,000	**10 indel<br>5 mismatch**	$(30, 3, 5)$	71.85	74.32	2,992	**96.64**	**96.92**	10,067

6 Commands: Tables of Commands and Parameters for Aligners

Commands used in our simulations:

- Bowtie2 *command*:

  bowtie2 -k <*list size*>{–fast or –sensitive} –local –score-min C,<*score*>,0 -p 24 -x human reads.fq -S test.sam

- BWA *command*:

  bwa bwasw -a 2 -b 6 -q 5 -r 3 -z <*sensitivity*>-t 24 human reads.fq

- Seqalto *command*:

  seqalto_basic align human.sidx -a -1 reads.fq -p 24

- Blasr *command*:

  blast reads.fq human.fa -nproc 24

Supplementary Table 7: Aligner commands

**Aligner**	**Number of threads**	**Other options**
Bowtie2	24	-k <*list size*>(if used)<br>-L 20<br>-N 1<br>–local<br>–score-min C,<*score*>,0 (if used)
BWA-SW	24	-a 2<br>-b 6<br>-q 5<br>-r 3<br>-z <*sensitivity*>
Seqalto	24	-
Blasr	24	-

Simulation results for these aligners are presented in Supplementary Tables 8-11.

Selecting Bowtie2 score parameter

In order to achieve the best recall performance, one needs to set the parameters of Bowtie2 in an optimal way. We first find the appropriate value for score. Let p_M and p_G be the error rates for mismatch and indel rates, respectively. A fragment of length ℓ has $p_M \times \ell$ and $p_G \times \ell$ mismatch and indel bases on the average, respectively. We set the score in such a way that most of the fragments can be handled by Bowtie2, probabilistically. Since the variance of the number of mismatches and indels within a fragment of length ℓ are $(1-p_M)p_M\ell$ and $(1-p_G)p_G\ell$, respectively, we set the score parameter as,

$$score = S_M \times (\ell - n_M - n_G) - S_{MM} \times n_M - S_G \times n_G$$

where S_M is match score in the used aligner, and S_{MM} and S_G denote mismatch and gap penalties, respectively. Also, n_M and n_G denoting the average and variance of the number of mismatches and indels computed, respectively, by

$$n_M = p_M\ell + t\sqrt{p_M(1-p_M)\ell}$$

and

$$n_G = p_G\ell + t\sqrt{p_G(1-p_G)\ell}.$$

The parameter t is a constant that is set to be 3 at low error rates and 1 or 2 at high error rates.

Supplementary Table 8: Bowtie2 simulation results-I.

L	ϵ%	T (sec)	R (%)	P (%)	Commands or Parameters
300	5	344	97.54	97.57	default
300	5	426	89.63	96.99	*score* = 340
300	5	422	92.19	96.56	*list size* = 2, *score* = 340
300	5	528	97.35	98.02	*list size* = 10, *score* = 340
300	5	368	97.06	97.08	*list size* = 10, sensitive
300	5	302	91.88	93.78	*list size* = 10, fast
300	5	960	98.58	98.71	*list size* = 40, *score* = 340
500	5	1,136	98.58	98.58	default
500	5	886	91.15	97.88	*score* = 624
500	5	894	93.90	97.65	*list size* = 2, *score* = 624
500	5	1,652	98.28	98.77	*list size* = 10, *score* = 624
500	5	1,662	98.51	98.51	*list size* = 10, sensitive
500	5	1,210	95.72	96.27	*list size* = 10, fast
500	5	4,712	99.24	99.27	*list size* = 40, *score* = 624
1,000	2	6,684	99.36	99.36	default
1,000	2	3,992	92.76	98	*score* = 1,364
1,000	2	2,626	92.61	99.20	*score* = 1,682
1,000	2	4,724	94.72	97.48	*list size* = 2, *score* = 1,364
1,000	2	3,020	95.22	99.13	*list size* = 2, *score* = 1,682
1,000	2	19,006	98.33	98.73	*list size* = 10, *score* = 1,364
1,000	2	7,030	98.74	99.65	*list size* = 10, *score* = 1,682
1,000	2	14,120	99.58	99.58	*list size* = 10, sensitive
1,000	2	9,212	99.36	99.36	*list size* = 10, fast
1,000	2	74,066	99.29	99.30	*list size* = 40, *score* = 1,364
1,000	2	21,744	99.75	99.84	*list size* = 40, *score* = 1,682

Supplementary Table 9: Bowtie2 simulation results-II.

L	ϵ%	T (sec)	R (%)	P (%)	Commands or Parameters
1,000	5	7,262	99.14	99.14	default
1,000	5	6,860	92.71	97.73	*score* = 1,028
1,000	5	3,620	92.54	98.81	*score* = 1,352
1,000	5	9,394	94.63	97.11	*list size* = 2, *score* = 1,028
1,000	5	4,722	95.16	98.71	*list size* = 2, *score* = 1,352
1,000	5	32,528	98.21	98.48	*list size* = 10, *score* = 1,028
1,000	5	19,060	98.79	99.42	*list size* = 10, *score* = 1,352
1,000	5	13,736	99.12	99.12	*list size* = 10, sensitive
1,000	5	8,606	98.27	98.32	*list size* = 10, fast
1,000	5	108,620	99.10	99.11	*list size* = 40, *score* = 1,028
1,000	5	71,968	99.64	99.69	*list size* = 40, *score* = 1,352
1,000	10	7,658	97.52	97.79	default
1,000	10	8,586	91.31	97.42	*score* = 600
1,000	10	11,156	93.74	96.67	*list size* = 2, *score* = 600
1,000	10	36,792	97.93	98.17	*list size* = 10, *score* = 600
1,000	10	13,844	96.84	97.06	*list size* = 10, sensitive
1,000	10	7,416	80.32	88.79	*list size* = 10, fast
1,000	10	119,864	98.87	98.88	*list size* = 40, *score* = 600
1,000	10 indel	7,882	98.33	98.45	default
1,000	10 indel	10,756	87.11	95.6	*score* = 200
1,000	10 indel	12,616	88.91	92.82	*list size* = 2, *score* = 200
1,000	10 indel	41,506	95.81	96.04	*list size* = 10, *score* = 200
1,000	10 indel	15,090	97.69	97.79	*list size* = 10, sensitive
1,000	10 indel	7,638	84.84	91.88	*list size* = 10, fast
1,000	10 indel	129,186	97.65	97.70	*list size* = 40, *score* = 200

Supplementary Table 10: BWA simulation results.

L	ϵ %	T (sec)	R (%)	P (%)	Commands or Parameters
300	5	141	96.4	96.5	z=2
300	5	517	97.8	97.83	z=10
300	5	1,754	97.28	97.3	z=40
500	5	221	97.29	97.32	z=2
500	5	875	98.37	98.39	z=10
500	5	3,172	96.92	96.93	z=40
1,000	2	474	98.55	98.54	z=2
1,000	2	1,752	98.91	98.92	z=10
1,000	2	11,513	97.61	97.62	z=40
1,000	5	370	98.03	98.06	z=2
1,000	5	2,985	98.74	98.76	z=10
1,000	5	6,349	96.49	96.5	z=40
1,000	10	367	97.20	97.26	z=2
1,000	10	1,176	98.14	98.16	z=10
1,000	10	2,718	95.3	95.32	z=40
1,000	10 indel	385	97.01	97.16	z=2
1,000	10 indel	1,140	97.91	97.94	z=10
1,000	10 indel	2,462	94.87	94.9	z=40

Supplementary Table 11: Seqalto simulation results.

L	ϵ %	T (sec)	R (%)	P (%)
300	5	4,990	88.60	98.37
500	5	19,412	83.86	98.79
1,000	2	58,114	98.94	99.40
1,000	5	104,100	78.72	99.18
1,000	10	15,516	0	0
1,000	10 indel	13,254	0	0

7 Analysis: Number of Unique Reports

In this section, we compare the number of reads reported as unique reads by Meta-aligner and other aligners in different scenarios. We consider $L = \{300; 500; 1,000\}$ bps with $\epsilon = \{2; 5; 10\}\%$ sequencing error rates. In addition, we consider the $\epsilon = 10\%$ indel-only case for $L = 1,000$ bps. Results are shown in Supplementary Figures 10-11. Simulation results for these cases are presented in these figures as follows: (1) Bowtie2 in different scenarios (in the default mode, fixed score option and default list size, fast mode with list size of 10, sensitive mode with list size of 10, fixed score option and list sizes of 2, 10 and 40), (2) Seqalto in default mode, (3) BWA-SW with $z = 2,\ 10,$ and 40, and (4) Meta-aligner.

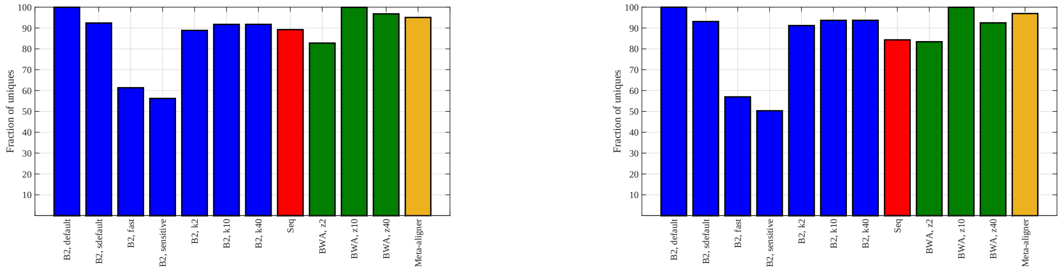

(a) $(L, \epsilon) = (300, 5\%)$, $(\ell, d) = (40, 2)$

(b) $(L, \epsilon) = (500, 5\%)$, $(\ell, d) = (40, 2)$

Supplementary Figure 10: **Number of unique reports for** $\epsilon = 5\%$. This figure shows number of unique reports for different aligners for read lengths of $L = \{300; 500\}$ bps and error rates of $\epsilon = 5\%$. Meta-aligner parameters (ℓ, d) are shown at the bottom of each figure (all with no slide). Also, Bowtie2 in the default mode, fixed score option and default list size, fast mode with list size of 10, sensitive mode with list size of 10, fixed score option and list sizes of 2, 10 and 40, Seqalto in default mode, and BWA-SW with $z = 2$, $z = 10$ and $z = 40$ results are presented.

Results show that Meta-aligner reports very large subset of mapped reads uniquely to the reference genome. Note that since Bowtie2 in default mode and BWA-SW with $z = 10$ reported only the best locations for all reads, they report a high percentage of uniquely mapped reads (more than Meta-aligner). However, Meta-aligner demonstrates better performance in terms of both execution time and precision compared to these two aligners at the aforementioned settings (Supplementary Sections 5-6). Also, in most cases, its recall rate is quite close to Bowtie2.

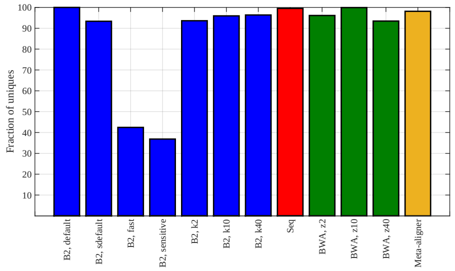

(a) $(L, \epsilon) = (1,000, 2\%)$, $(\ell, d) = (40, 1)$

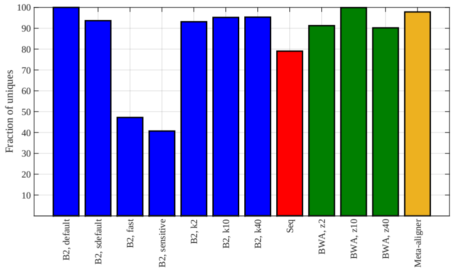

(b) $(L, \epsilon) = (1,000, 5\%)$, $(\ell, d) = (40, 2)$

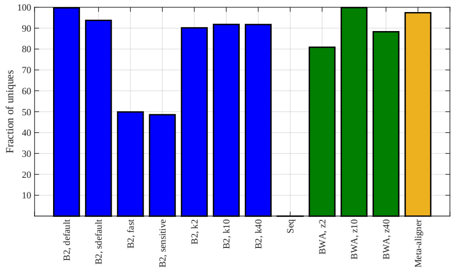

(c) $(L, \epsilon) = (1,000, 10\%)$, $(\ell, d) = (30, 3)$

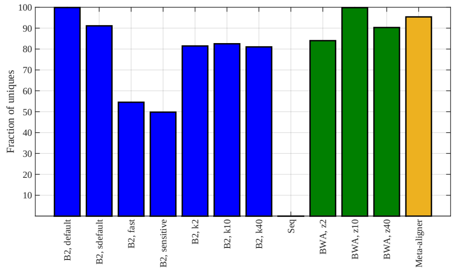

(d) $(L, \epsilon) = (1,000, 10\%)$ (only indel), $(\ell, d) = (30, 2)$

Supplementary Figure 11: **Number of unique reports for $L = 1,000$.** This figure shows number of unique reports for different aligners for read length of $L = 1,000$ and different error rates of $\epsilon = \{2; 5; 10\}\%$. Meta-aligner parameters (ℓ, d) are shown at the bottom of each figure (all with no slide). Also, Bowtie2 in the default mode, fixed score option and default list size, fast mode with list size of 10, sensitive mode with list size of 10, fixed score option and list sizes of 2, 10 and 40, Seqalto in default mode, and BWA-SW with $z = 2$, $z = 10$ and $z = 40$ results are presented.

8 Analysis: PacBio Input Reads

We use the published PacBio datasets via the following link released within the read archive NCBI GenBank SRX533609: "http://www.ncbi.nlm.nih.gov/sra?term=SRX533609". We use the first two files, SRR1304331 and SRR1304332. We merge these two files and convert them to *fastq* format by pbh5tools in "http://github.com/PacificBiosciences/pbh5tools", with command "python bash5tools.py <read name>.bas.h5 –outFilePrefix pacbioTest –outType fastq –minLength 500". Consequently, we will be using reads of length more than 500 and with all qualities. Thus, $174,537$ reads with different lengths (minimum length of 500 bps and maximum length of 39445 bps) are generated. For aligning PacBio reads, we first use PE algorithm of Meta-aligner to estimate values of ℓ and d and value of sliding. The PE algorithm estimates $(\hat{\epsilon}_{\mathbf{M}}, \hat{\epsilon}_{\mathbf{G}}) = (\mathbf{2.8\%}, \mathbf{13.8\%})$, **and propose** $(\ell, \mathbf{d}, \mathcal{S}_\mathbf{1}) = (\mathbf{25}, \mathbf{1}, \mathbf{0})$ by considering time and recall rate together, or $(\ell, \mathbf{d}, \mathcal{S}_\mathbf{1}) = (\mathbf{25}, \mathbf{2}, \mathbf{5})$ when **recall rate is significant**.

Simulation results show that **only first $\mathbf{6,000}$ bases of all reads are enough for the anchoring step**. This result is shown in Supplementary Figure 12. Also, in the following, we explain why such value of read length is theoretically enough for anchoring. This figure shows that if we use all bases of all reads, mapping percentage increases only by 2.54%, while anchoring time increases by 1440 sec and overall time of first stage increases by 2528 sec. Using only first i bases of all reads for anchoring in Meta-aligner can be controlled by -tr i command.

We now describe why 6 Kbps of reads is enough for anchoring. First, suppose noise is added to PacBio reads through an i.i.d. model. Note that Bowtie can not handle indels within a read, and only a single indel at the first or last bases of a read can be handled by Bowtie. Assume that number of mismatches and indels within a sub-fragment of length ℓ are denoted by $\mathcal{N}_M$ and $\mathcal{N}_G$, respectively. Thus, a sub-fragment of length ℓ with $d = 1$ is aligned to its true position with probability of

$$\begin{aligned} P_a &= \mathbb{P}\{\mathcal{N}_M = 0, \mathcal{N}_G \leq 1 \text{ and two sides bases}\} + \mathbb{P}\{\mathcal{N}_M = 1, \mathcal{N}_G = 0\} \quad (5) \\ &= (1 - \epsilon_M - \epsilon_G)^{\ell} + 2\epsilon_G (1 - \epsilon_M - \epsilon_G)^{\ell-1} + \ell\epsilon_M (1 - \epsilon_M - \epsilon_G)^{\ell-1} \\ &= (1 + (\ell - 1)\epsilon_M + \epsilon_G)(1 - \epsilon_M - \epsilon_G)^{\ell-1} \quad (6) \end{aligned}$$

where ϵ_M and ϵ_G are mismatch and indel error rates, respectively. If we take $\ell = 25$, $\epsilon_M = \hat{\epsilon}_M$ (with $\hat{\epsilon}_M = 0.028$) and $\epsilon_G = \hat{\epsilon}_G$ (with $\hat{\epsilon}_G = 0.138$), then $P_a = 0.0232$. If a read has N disjoint random ℓ-mers for $(\ell, d) = (25, 1)$, then the probability of alignment to true location of this read using the first stage of Meta-aligner is

$$P_t = 1 - (1 - P_a)^N - N \times P_a (1 - P_a)^{N-1}$$

For $N \geq 240$ (or $L \geq 6$ Kbps), $P_t \approx 0.976$. Thus, if a read has at least 240 disjoint random 25-mers, it is aligned to its true location with high probability. Also, note that for a length of 6 Kbps, only a very small fraction of repeats in the genome remain unbridged.

Now, we compare mapping rates for two different values of $d = 1$ and $d = 2$ with $\ell = 25$. Using $d = 2$ and only first $6,000$ bases of reads, mapping percentage increases by 3.23% while time of anchoring increases by 280 sec and overall time of first stage increases by $3,092$ sec. When $d = 2$ is used, extracted fragments of length ℓ from reads that contain indels within their first or last bases can be aligned by Bowtie. This is one reason that the mapping percentage increases in this case.

We also compare results when using slide window for $\ell = 25$ and $d = 1$ *or* 2. Comparison of the mapping percentage and time of anchoring is presented in Supplementary Table 12. For maximum mapping percentage, we propose that user uses $\ell = 25$, $d = 2$ and slide value of 5 (using whole length or $L_{max} = 6$ Kbps) with mapping percentage of $\approx 85 - 87\%$. The overall Meta-aligner results for the PacBio input reads are shown in the Supplementary Table 13.

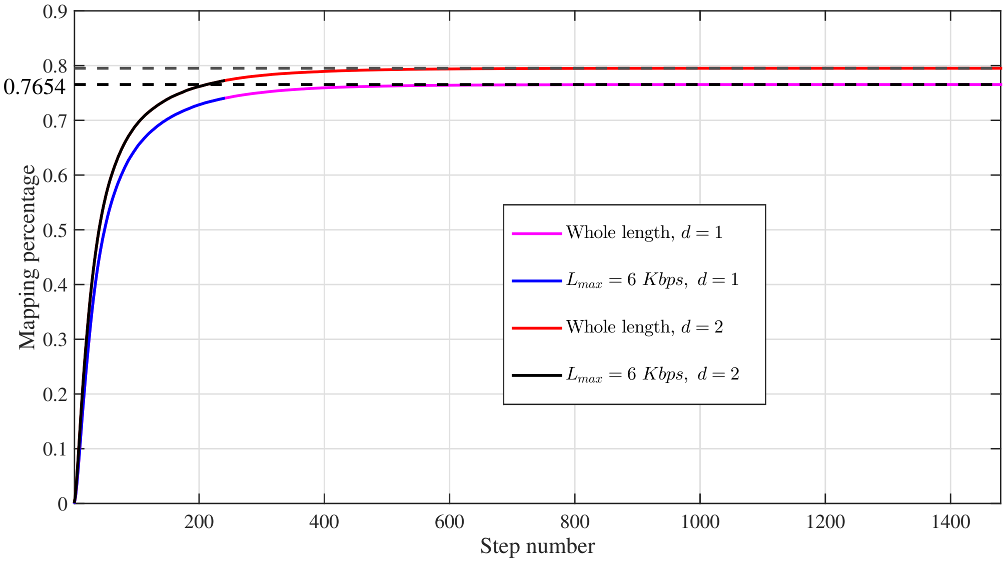

Supplementary Figure 12: **PacBio required length.** This figure shows that using only first 6 Kbps of all reads achieves the acceptable step-by-step mapping percentage. In this simulation, $(\ell, d, \mathcal{S}_1) = (25, 1, 0)$ is used.

Supplementary Table 12: PacBio parameter analysis at the first stage of Meta-aligner. M_1 shows mapping percentage of the first stage of Meta-aligner.

ℓ	d	$\mathcal{S}_1$	L_{max} (bps)	Anchoring time (sec)	M_1 (%)
25	1	-	6,000	886	74.00%
25	1	5	6,000	2,398	82.64%
25	2	-	6,000	1,165	77.23%
25	2	5	6,000	3,031	85.25%
25	1	-	whole	2,326	76.54%
25	1	5	whole	5,837	84.61%
25	2	-	whole	2,665	79.51%
25	2	5	whole	6,088	87.04%

Supplementary Table 13: Results of Meta-aligner for the generated PacBio read set. M_1 and M_{tot} show mapping percentages when only the first stage and two first steps of Meta-aligner are used, respectively. Also, T_1 and T_{tot} show running time of these two cases, respectively.

ℓ	d	$\mathcal{S}_1$	L_{max} (bps)	T_1 (sec)	M_1 (%)	T_{tot} (sec)	M_{tot} (%)
25	1	-	6,000	19,407	74	**21,525**	79.49
25	1	-	whole	21,935	76.54	24,160	88.99
25	2	-	6,000	22,499	77.23	29,399	91.29
25	2	-	whole	22,718	79.51	**25,453**	**92.15**
25	2	5	6,000	25,983	85.25	28,467	89.95
25	2	5	whole	27,582	87.04	39,041	**97.97**

9 Manual: Meta-aligner Commands

Usage

meta-aligner [options]* -x <index> -fa <fa> -r <r> -o [<hit>]

Main arguments

Meta-aligner main arguments and their descriptions are as follows,

-x <name>:

The base name of the indexes for the reference genome used at each step of the Meta-aligner. *Note that if any aligner is used at the alignment or assignment stages, their indexes must exist with this name.* For Soap2 and mrsFast aligners which are used at the first stage of Meta-aligner, this name must be used *without any suffix.*

-fa <name>:

The reference genome which is used for the local alignment (assumed to be in Fasta format).

-r <name>:

The base name of the input read set (assumed to be in FastQ format).

-o <name>:

File to write SAM alignments to. By default, alignments are written to "output.sam".

Options

Meta-aligner options are as follows,

Estimation option

User can choose PE algorithm for estimating required parameters as follows,

-est <1 or 2>:

By this command estimation is used and ℓ_1 (fragment length), d (maximum Hamming distance), mismatch and indel error rates and normalized cutting distance (-ed command) are estimated by Meta-aligner. Meta-aligner propose parameters for two scenarios, 1) recall rate or 2) time are significant. By setting -sig command, each of these scenarios are selected. Default mode of estimation is off.

Input option

User can choose input format as follows,

-FA:

Reads are Fasta files. Fasta files usually have extension .fa, .fasta, .mfa or .fnaor.

-pg:

Percent of gap within the input read set. This value is used in -ed options. The default value is 0.01. This parameter can be estimated when user uses the PE algorithm.

Alignment options

User can change alignment parameters for achieving better performance at the first stage (and/or the first step of the second stage) of the Meta-aligner.

-al <int>:

Flag for using different short read aligners at the alignment stage only. Flag values are: Bowtie 1 / mrsFast 2 / SOAP2 3. The default is Bowtie (1).

-l1 <int>:

The fragment size (ℓ_1) which is used at the alignment stage, and the first step of the assignment step. The default value is 40. This parameter can be learned when user uses the PE algorithm.

-sl1 <int>:

The length of sliding window. This parameter is used at the alignment stage and the first step of the assignment stage of Meta-aligner. The default value is 0, i.e., no sliding is used. This parameter can be learned when user uses the PE algorithm.

-cfd1 <int>:

The consecutive distance between two anchored fragment which is used for confirming two fragments of a read and anchor read ($\mathscr{G}_1$). This parameter is used at the alignment stage and the first step of the assignment stage of Meta-aligner. The default value is $\mathscr{G}_1 = 0.1\ell_1$.

-d <int>:

Edit distance between fragments and the reference genome using for alignment. This parameter is used at the alignment stage and the first step of the assignment stage of Meta-aligner. Setting this value to zero means that only exact matches are desired. The default value is 2. This parameter can be learned when user uses the PE algorithm.

- For Bowtie: this command works as -v (may be an integer from 0 through 3) and determines only number of mismatches.
- For mrsFast: this command works as -e.
- For Soap2: this command works as -v.

-tr <int>:

Length of reads that are trimmed and only <int>bases of each read is used for anchoring at the alignment stage and the first step of the assignment stage of Meta-aligner. The remaining bases of each reads are used in the local alignment. In the default mode, this value is not used.

Assignment options

User can change these parameters for achieving better performance at the second stage of Meta-aligner.

-l2 <int>:

The fragment size (ℓ_2) which is used at the second step of the assignment stage. The default value is 150.

-sl2 <int>:

The length of sliding window for the second step of the assignment stage. The default value is 50.

-cfd2 <int>:

The consecutive fragments distance which is used for confirming two fragments of a read and anchor it ($\mathscr{G}_2$) which is used at the second step of the assignment stage. The default value is $\mathscr{G}_2 = 0.1\ell_2$.

-seedmm2 <int>:

Number of mismatches which is allowed in a seed alignment at the second step of the assignment stage. The default value is 1.

-seedlen2 <int>:

Length of the seed substrings to align at the second step of the assignment stage. The default value is 20.

-ls1 <int>:

List size of the assignment stage when Bowtie is used (at the second step of the assignment stage of Meta-aligner). The default value is 10.

-ls2 <int>:

List size of the assignment stage when Bowtie2 is used (at the third step of the assignment stage of Meta-aligner). The default value is 40.

-thrsc <double>:

Threshold of path selection step at the assignment stage of Meta-aligner (for both Bowtie and Bowtie2). Paths are filtered by their scores. The default value is 0.3.

Scoring options

User can change the desired score for local alignment step of Meta-aligner.

-ms <double>:

The match score.

-mp <double>:

The mismatch penalty.

-gp <double>:

The gap penalty.

Reporting options

User can choose the desired report of the Meta-aligner.

-dis:

This option discards local alignment of the anchored reads. By using this option, only reads, their flags and positions on the reference genome are reported. This parameter is only used at the first stage of Meta-aligner.

-disHeader:

This option suppresses the header of the output SAM file.

Other options

Meta-aligner provides some options such that users can match Meta-aligner with their platform.

-step <{1 or 2 or 3}>:

This parameter specifies that Meta-aligner is run up to the selected *step*, in case of selecting "1": run only the alignment stage; "2": run the alignment stage and the first step of the assignment stage; "3": run all steps of Meta-aligner. The default value is "2".

-dir <address>:

If this parameter is used, Meta-aligner creates a new directory at the input address, and all steps are executed at that address. The default address is "./results".

-p <int>:

Number of threads which is used for running Meta-aligner (both stages). The default value is 1.

-ed <double>:

This parameter controls the normalized cutting length of the local alignment table in the Smith-Waterman algorithm (relative to each read length). With this parameter, only $\{ed/2\times$ read length$\}$ cells adjacent to the original diagonal of the local alignment table are used for local alignment procedure. This parameter must be between 0 (consider only original diagonal cells of the dynamic table) and 2 (consider all cells of the dynamic table). The default value is $5 \times pg$. This parameter can be estimated from indel rate when user uses the PE algorithm.

-ram <double>:

User can set the available RAM when running Meta-aligner. By this parameter, user can run Meta-aligner in all platforms without any restriction of RAM. By using this command, Meta-aligner handles number of threads (-p) and length of reads at the local alignment step. If some reads cannot be processed by this value of RAM (even with one thread), Meta-aligner reports these reads in a file (named "NotEnoughRAM.txt") which consists of reads in Fastq format, with their flags and the anchored positions written in their header section by underline.

-h:

Used for printing of Meta-aligner commands.
